# Risk Factors Disrupting Wholistic Wellness Among Indigenous Families During COVID‐19

**DOI:** 10.1111/famp.70107

**Published:** 2026-02-07

**Authors:** Kya Locklear, Kristi Ka'apu, Catherine E. O'Connor, Michelle Johnson‐Jennings, Juliette Rau

**Affiliations:** ^1^ Tulane University School of Medicine New Orleans Louisiana USA; ^2^ Tulane University School of Social Work New Orleans Louisiana USA; ^3^ Indigenous Wellness Research Institute University of Washington Seattle Washington USA

**Keywords:** COVID‐19, indigenous, resilience, risk, wellness

## Abstract

The impact of the COVID‐19 virus disproportionately affected U.S. Indigenous peoples, who experienced the highest infection and death rates in comparison with non‐Indigenous peoples. In this article, we use the framework of historical oppression, resilience, and transcendence (FHORT) to understand how Southeastern Indigenous peoples in the United States navigated hardships associated with the COVID‐19 global pandemic. This culturally congruent framework contextualizes imbalances found at individual, family, and community ecological levels, illustrating a direct correlation to sociopolitical, historical, and cultural oppression. This research assessed interconnections of structural inequity and associated disruptions to Indigenous wholistic wellness amid the pandemic. Thirty‐one community‐based, critical ethnographic interviews were conducted following an Indigenous toolkit for ethical and culturally sensitive research to understand quantitative risk factors associated with participant responses to COVID‐19. The following themes emerged: (a) racism, sexism, and discrimination; (b) increased trauma, financial stress, and violence; (c) physical symptoms; (d) impaired unity; and (e) disintegrated support and kinship networks. Risk factors associated with COVID‐19 emerged in large part from systemic inequity, incongruence between Indigenous family values and physical distancing protocols, and impaired collectivism. Future crisis interventions should promote traditional protective factors to offset the impact of historical oppression, consistent with the FHORT.

## 1 Introduction

Indigenous Peoples of the United States (U.S.) experience some of the most widely documented health disparities and trauma—including but not limited to physical and mental health and violence—compared to any other ethnic group (Black et al. 2011; Burnette and Figley 2017). The scope of this inquiry is limited to American Indians and Alaska Natives (Indigenous peoples), who represent over 5 million people in the U.S. and can be found across 574 federally recognized sovereign tribes and more than 100 state‐recognized tribes (Congressional Research Service 2024). Federally recognized tribes have tribal sovereignty and treaty agreements in which the U.S. recognizes these Nations as having inherent sovereignty and sovereign powers (Joseph 2023) and the U.S. government is responsible for the health and well‐being of these Indigenous people (U.S. Commission on Civil Rights 2004). Despite this legal responsibility, Indigenous peoples continue to experience severe victimization stemming from historical trauma and oppression and through ongoing settler colonial efforts to eliminate Indigenous identity (Black et al. 2011; Emerson and Montoya 2021; Walters, Beltran, et al. 2011; Walters, Mohammed, et al. 2011). COVID‐19 has highlighted these disparities and their associated risks further.

### 1.1 U.S. Indigenous Peoples and COVID‐19

Indigenous peoples continue to be burdened excessively by infectious disease due to socioeconomic determinants shaped by coloniality and culture/land loss, including but not limited to high rates of poverty, inadequate food and water access, under‐funded health care systems, and high comorbidity and disease diagnoses (Blue Bird Jernigan et al. 2020; Lee et al. 2023). Literature established the disproportionate impact of COVID‐19 on Indigenous peoples, who experience the highest infection and death rates compared to other racial/ethnic groups in the U.S. (Goldman and Andrasafay 2022; Hornback and Ramos 2021; Leggat‐Barr et al. 2021). U.S. Indigenous peoples were hospitalized at rates 4 times higher than Whites (Goldman and Andrasafay 2022), and those living on reservations experienced a mortality rate 2.8 times higher than Whites (Leggat‐Barr et al. 2021). COVID‐19 widened the life expectancy gap between Indigenous and non‐Hispanic White persons, with an average of 4.5 lost years in 2020 and 6.4 in 2021 (Goldman and Andrasafay 2022).

Indigenous communities experienced unprecedented and unequal COVID‐19 devastation, yet scarce academic research has analyzed risk factors for pandemic‐related impacts related to imposed historical social vulnerability (Leggat‐Barr et al. 2021; Yellow Horse and Huyser 2022). Further, understanding COVID‐19 among Indigenous peoples must acknowledge past collective threats to their survival and center Indigenous knowledge systems that allow Indigenous peoples to adapt and thrive in physical and psychosocial health threats (Elliott‐Groves et al. 2020).

Even amid high rates of COVID‐19, resilience was common among Indigenous peoples. Protective factors arose when Indigenous values prevailed, including exercising tribal sovereignty by closing casinos and tribal nation borders purposefully, practicing cultural traditions when social distancing, having strong family and community ties, and mobilizing communities for high vaccination rates (Connolly et al. 2021; Evans et al. 2022; Foxworth et al. 2021; Hunter et al. 2022). COVID‐19 further intensified health disparities for minoritized people (Fortuna et al. 2020), exposing the need to decolonize from systemic practices that drive these injustices. Decolonization involves challenging colonial assumptions of hierarchy, superiority, and self‐orientation in favor of Indigenous values, such as collectivism, which were efficacious for Indigenous peoples during the pandemic as communities made difficult decisions for the good of the whole (Blume 2022).

### 1.2 Framework of Historical Oppression, Resilience, and Transcendence

Grounding this inquiry, the framework of historical oppression, resilience, and transcendence (FHORT) is a culturally congruent framework that situates barriers to Indigenous health and prosperity as consequences of settler colonial historical oppression. Historical oppression explicates past and present oppression and settler colonial injury, or systematic erasure of Indigenous peoples and ideologies to replace them with settler societies that are embodied and imposed on the daily lives of Indigenous peoples socially and epigenetically (Avalos 2021; Burnette and Figley 2017). Past and present experiences of historical oppression include genocidal acts and policies, removal from lands, compulsory boarding schools, and prohibition of spiritual and cultural practices, which have inter‐ and multi‐generational impacts that disparately worsen the health of Indigenous Peoples (Evans‐Campbell 2008). Colonization drives structural and institutional inequities that create inadequate and unequal access to promotive health conditions (Leung et al. 2022). The FHORT provides a necessary strength‐based framing of Indigenous resilience and emphasizes Indigenous survivance, or how Indigenous knowledge and lifeways prevail through active resistance to colonialization, such as commitment to homeland (Burnette and Figley 2017; Vizenor 2008).

Complementary to the FHORT is Indigenous *wholism*, defined as Indigenous spiritual, emotional, mental, and physical well‐being through “reciprocal interconnections of self, individual, family, community, nation, society, and creation” (Absolon 2010, 25). Healing and resilience require balanced emotional, mental, physical, and spiritual human growth maintained by relationships to society, land, and the spiritual realm, yet settler colonial erasure and marginalization of Indigenous identity fuel imbalances among Indigenous Peoples' lives (Absolon 2010; McCabe 2008). Balance, or lack thereof, among risk and protective factors across multiple levels informs one's wellness and ability to thrive (Burnette and Figley 2017).

Historical oppression is incongruent with Indigenous wholism, values, and relationality, which refers to the Indigenous worldview that emphasizes reciprocal relationships between self, family, community, the natural world, and the spiritual realm (Elliott‐Groves et al. 2020; Wildcat and Voth 2023). Historical oppression threatens the well‐being of Indigenous Peoples and traverses all socioecological dimensions of health: (a) individual (e.g., suicidal ideation, substance misuse, chronic and traumatic stress), (b) relational/familial (e.g., intimate partner violence, disrupted family communication patterns), (c) community/cultural (e.g., cultural disorientation and disruption, displacement from traditional lands, threats to tribal sovereignty), and (d) societal (e.g., systemic racism, marginalization; Burnette and Figley 2017; Christensen and Damon 2022; Evans‐Campbell 2008; McKinley and Lilly 2022). Thus, historical and contemporary assaults inform the collective health Indigenous peoples experience during and independent of COVID‐19.

Understanding the impact of COVID‐19 on Indigenous families is critical, as the pandemic exacerbated pre‐existing health and social disparities rooted in historical trauma, systemic inequities, and structural violence (Black et al. 2011; Emerson and Montoya 2021; Walters, Beltran, et al. 2011). Indigenous communities faced disproportionate rates of infection, economic hardship, and disruptions to cultural and kinship networks, intensifying challenges to overall well‐being (Hatcher et al. 2020; Power et al. 2020). The FHORT framework is particularly relevant in this context, as it provides a holistic lens to examine how historical oppression, resilience, and structural factors intersect to shape Indigenous family experiences. By applying FHORT, this study not only captures the immediate effects of COVID‐19 but also situates these experiences within broader historical and systemic contexts. Integrating these perspectives strengthens the study's foundation, underscoring the necessity of understanding COVID‐19's impact through a framework that acknowledges both historical trauma and resilience.

### 1.3 Current Study

This study explored risk factors for disrupted resilience and imbalanced health among members of one southeastern tribe during COVID‐19 through the FHORT. Specifically, the overarching research question guiding this inquiry was: *How does COVID‐19 relate to individual‐ and communal‐level risk factors for Indigenous women?* The FHORT provides a holistic lens to examine these questions, situating pandemic‐related challenges within broader historical and systemic contexts. This framework allows us to capture the immediate effects of COVID‐19 while also highlighting how historical oppression and resilience intersect to shape Indigenous family experiences.

## 2 Method

### 2.1 Research Design

This study served as a supplemental component of a larger critical ethnography (McKinley and Theall 2021; McKinley et al. 2023) and its purpose was to understand how Indigenous families were affected by the COVID‐19 pandemic. This study is complementary to separately published investigations of family dynamics among this population during COVID‐19, including topics of family coping, loss and grief, and caregiving (Glover et al. 2025; Ka'apu et al. 2024, 2025). We aimed to explore the experiences of Indigenous families in the southeastern U.S. through the perspectives of 31 heads of household women and focused on how risk factors influenced the responses of individuals, families, and communities to stressors during the crisis.

A critical ethnographic approach was utilized, prioritizing empowering participants' voices in the dissemination of knowledge, making it an appropriate continuation of longstanding community‐based participatory research with this tribal community (Burnette et al. 2014; Carspecken 1996). Participants were recruited from the eight communities that make up a southeastern reservation of a U.S. federally recognized tribe and were recruited until thematic saturation was reached, which occurred at 31 individuals. Saturation was determined when no new themes or insights emerged from additional interviews, indicating that sufficient data had been collected to comprehensively address the research questions (Guest and MacQueen 2008). Participants' and tribal communities' identifiable information is kept confidential in compliance with agreements made with the tribal agreements and following protocols for ethical and culturally sensitive research (Burnette et al. 2014).

### 2.2 Data Collection

All procedures performed in this study involving human participants were in accordance with the ethical standards of the institutional and/or national research committee and with the 1964 Helsinki Declaration and its later amendments or comparable ethical standards. This research gained approval from the social‐behavioral sciences component of Tulane University's IRB board [Study: 2018‐1372‐OTH]. After attaining approval from Tulane University's Institutional Review Board and tribal approval for the study, participants were recruited by word of mouth, posting flyers online and in person as well as working with agency heads and cultural insiders. Written informed consent was obtained from all participants, who were then interviewed by the principal investigator (PI) or a tribal research team member, with participants given the option to choose the interviewer. Inclusion criteria included being a part of the focal tribe, female, and head‐of‐household. The decision to focus on women's experiences was guided by both cultural and methodological considerations. The community advisory board, consisting of Indigenous leaders and stakeholders, recommended centering the voices of women, as they are often the heads of households and key decision‐makers within their families and communities. Additionally, the tribe in our study has a historically matrilineal and matrilocal structure, where women play a central role in maintaining family cohesion, cultural traditions, and community resilience. Interviews were conducted in December 2021 and April 2022 and ranged from 23 to 85 min in length.

A semistructured interview guide, developed with Indigenous cultural insiders, was utilized to assess participants' past and present experiences of COVID‐19, addressing topics related to physical, mental, and spiritual changes among individuals and families and social changes in the broader community (Burnette et al. 2014; Carspecken 1996). Examples of relevant questions include: What have been the hardest things about the COVID‐19 pandemic for you? How have you made it through these times? The scope of this inquiry was limited to women who were head‐of‐household, with past and present perspectives of the COVID‐19 global pandemic (see Table 1 for additional demographics). Participants were given $50 USD via Clincard, a reloadable debit card. The demographics of the participants closely reflect those of the women in the tribe, ensuring that the findings are representative of the broader female population within the community.

**TABLE 1:** Participant demographics (*N* = 31).

	*n* (%)
Age (range 29–56)	39.79 (SD = 6.34)
Community of residence	
Main tribal community	14 (45.16%)
Other tribal communities	17 (54.83%)
Highest level of education	
Some high school	3 (9.67%)
High school graduate or equivalent	4 (12.90%)
Some college	8 (25.80%)
Associate degree	6 (19.35%)
Bachelor's degree	5 (16.13%)
Master's degree	3 (12.90%)
Employment	
No employment	7 (22.58%)
Part‐time employment	1 (3.23%)
Full‐time employment	23 (74.19%)
Relationship status	
Single	12 (38.70%)
In a relationship, not married	6 (19.35%)
Married	9 (29.03%)
Widowed	2 (6.45%)

### 2.3 Data Analysis

Data analysis followed a rigorous, team‐based approach using pragmatic horizon analysis (Carspecken 1996), a critical theory‐informed method that identifies subjective, objective, and intersubjective meanings in the data. Themes were identified through a theoretically driven and inductive approach, guided by the FHORT framework while allowing emergent themes to surface from participant narratives. The coding team consisted of two Indigenous graduate students, two additional graduate research assistants trained in qualitative methods, and tribal community members who served as cultural consultants. To ensure consistency, the team underwent training on the FHORT framework, Indigenous research methodologies (e.g., community‐based participatory approaches that emphasize relational accountability, cultural protocols, and Indigenous ways of knowing; Burnette et al. 2014), and collaborative coding exercises. Data were analyzed using NVivo qualitative analysis software. The PI established an initial coding hierarchy, while three trained analysts conducted line‐by‐line coding, verifying each other's work. The PI reviewed coding outputs and facilitated resolution of discrepancies through discussion with the coding team. Consensus coding was employed rather than formal calculation of interrater reliability statistics, a process aligned with collaborative and culturally congruent qualitative practices (Guest and MacQueen 2008). Indigenous informants played a key role in reviewing preliminary themes, providing cultural insights, and ensuring interpretive accuracy. During member checks, participants received their transcripts and were invited to provide feedback on the final themes. A detailed summary of identified themes, including illustrative quotes, was shared with participants, cultural insiders, and the tribal council.

### 2.4 Positionality Statement

Our research team brings diverse perspectives rooted in Indigenous identity, lived experience, and long‐standing partnerships with Indigenous communities. Three of the five co‐authors are Indigenous scholars who ground their research, practice, and teaching in Indigenous knowledge systems, relational accountability, and commitments to strengthening the health and well‐being of Indigenous peoples. Their lived experiences and community connections guide the framing, analysis, and interpretation of this work. The PI, while not Indigenous, has worked in partnership with Indigenous tribes and communities for over a decade. She is committed to allyship and has cultivated respectful, reciprocal research relationships that center Indigenous voices, sovereignty, and cultural frameworks. Together, our team draws on both Indigenous and allied perspectives to uphold cultural humility, prioritize community benefit, and contribute to research that advances equity, resilience, and healing for Indigenous peoples.

## 3 Results

**FIGURE 1:**
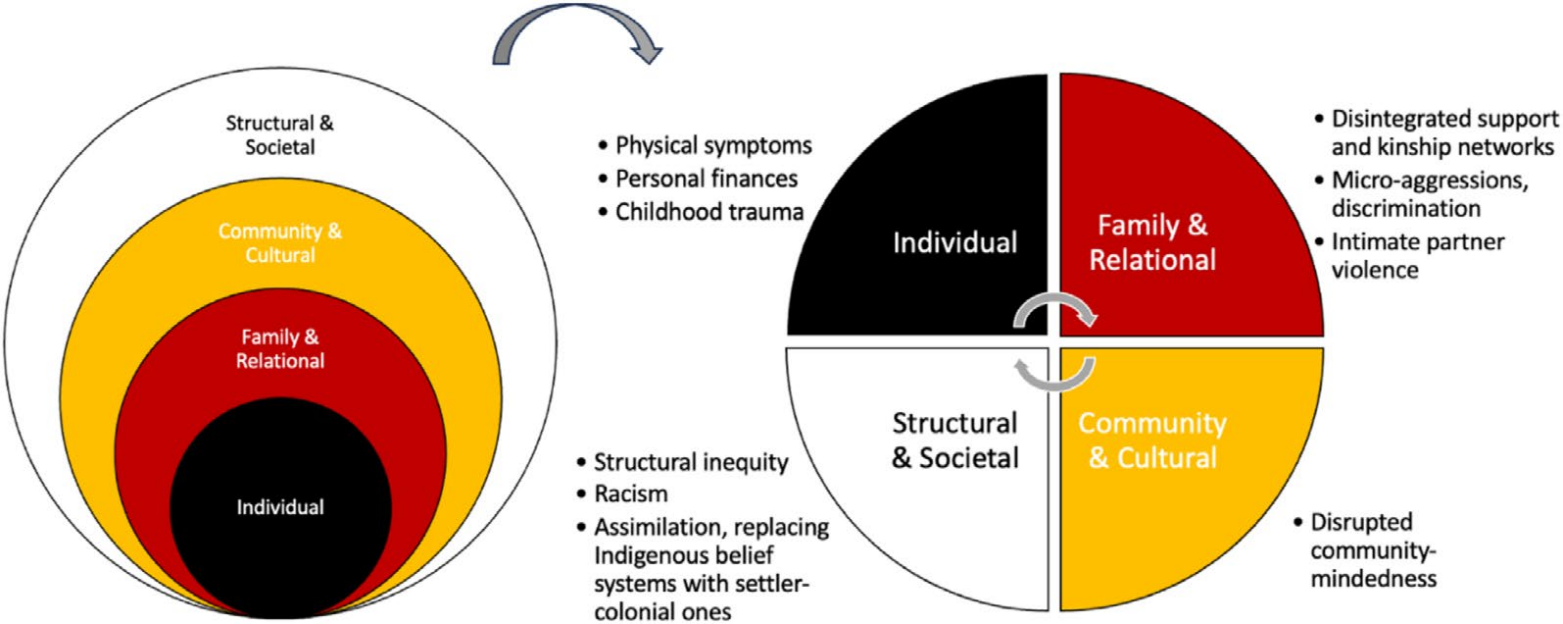
COVID‐19‐Related Risk Factors Through Ecological Levels of the FHORT. Within themes, emergent risk factors can be arranged in accordance with ecological levels of the FHORT for wholistic analysis. Interactions among risk factors are bidirectional and cyclical, and the existence of these factors with promotive ones informs how and if one experiences health and resilience.

Resiliency and adaptability when experiencing crises related to COVID‐19 was displayed; yet risk factors emerged from exacerbated inequities related to historical oppression that strained resources and supports. Risk factors, noted by all participants (*N* = 31), were often complex and fell across all ecological levels of the FHORT and arranged roughly from individual to structural and societal; see Figure 1. The following themes emerged: (a) individual experiences of racism, sexism, and discrimination; (b) personal exposure to trauma, financial stress, and violence; (c) physical symptoms at the individual level; (d) structural challenges such as impaired unity; and (e) societal‐level disintegration of support and kinship networks. All names used in the results section are pseudonyms to protect participant confidentiality.

### 3.1 Racism, Sexism, and Discrimination: “They're Being More Vocal About Being Racist”

Racism was a ubiquitous societal norm, frequently perpetuated as an interpersonal, daily experience. Some participants experienced discrimination across their lifetime, and others said the pandemic may have heightened their awareness of discrimination. Andrea thought the pandemic increased her awareness of racism toward Indigenous peoples, particularly on social media, saying, “I was blind to it before.” She continued, “You start to notice little comments that people make. And a lot of people that were silent about racism or being racist. They're being more vocal about being racist now.” Some participants felt they had experienced discrimination for many years, but it was more noticeable, or worse, during the pandemic. Pamala expressed: “They were blaming the Natives [Indigenous peoples] for bringing in COVID‐19. When they try to blame people, instead of trying to think of a solution, they are creating a problem.”

Many participants explained they had experienced discrimination long before the pandemic. Luz shared:

In the early 70s, we were considered colored, so we were not allowed to eat in front of the restaurant. We, as Native Americans, had to go to the back of the restaurant to eat. I experienced that as a little girl.

Some participants felt discrimination had become so ingrained in their day‐to‐day lives, it had become expected or normalized. When asked how this may impact her, Natasha answered:

The sexism and racism, I've had to deal with for so long. It's just become almost normal. I've had people come up to me [and ask], “Do you still live in teepees?” Or I've had this older White gentleman ask me, “Well, how does it feel not to pay taxes?” I kind of laughed it off because we still pay tax. I didn't know what he was talking about.

Victoria explained she observed these attitudes in her day‐to‐day life as well, sharing:

It's changed as far as how blatant [the discrimination] is. “I'm going to say or do or treat you the way I want to treat you.” What is that, they don't have any censor? Cashiers and the people at the fast‐food places have the attitude of “I don't like you. I don't want you here. I want a job, but I don't want you here,” you know?… So, the heightened level of discrimination, yes, it has heightened a lot.

Racism, sexism, and discrimination were experienced during the pandemic and beyond and embedded in the lives of these participants.

### 3.2 Increased Trauma, Financial Stress, and Violence: “It Was Pretty Stable…Until 2020”

Financial instability increased for some during the pandemic. Jenna explicated financial strain when COVID‐19 exposure rates increased, sharing, “Whenever the numbers started going up again this past January, my food sales weren't doing as good, so I kind of got behind on the car note.” She stayed steadfast, telling her children, “Don't worry, we've been here before. No car, no money, so we've been here before, and we've come out of it. We'll come out of it again.”

The pandemic created circumstances where trauma and violence were more common. As Molly shared, after her ex‐husband lost his job, he started drinking more heavily, impacting their coparenting relationship and his relationship with their children. Molly explained:

He's a mean drunk. So, that affected us negatively. Our coparenting relationship wasn't good. And I believed at one point he was trying to brainwash my kids. like putting things in their heads. I think that was more so the drinking and him being drunk. It was [a] pretty stable coparenting relationship, until 2020.

For others, the pandemic exacerbated existing stress in relationships. For Jenna, financial troubles, her ex‐boyfriend's drug use, and trust issues culminated in intimate partner violence. She shared:

We were already stressing out with everything. Whatever little money we did have, he would go blow it on drugs. I said, “I can't keep doing this. The babies need diapers. We need gas.” Thank God I was getting food stamps, it basically helped keep my kids eating… One night I know he pulled a knife out. I never knew what was the reason of it, and when I asked, he was like, “Oh, you're crazy, I wasn't gonna do nothing to you. I just pulled it out to scare you.” So, I wouldn't sleep for the fact that I was like, “Is he in his right state of mind or is he not?”… I got to where I would start sleeping with my other kids just to make sure nothing happens to my other kids.

Some participants said trauma and violence occurring in childhood or the years leading up to the pandemic impacted their response to COVID‐19. Some, like Monique, said the pandemic opened old wounds. Monique shared:

I really don't wanna [*sic*] feel how I felt back in December. I know that place and I don't ever wanna [*sic*] go back. I actually grew up with my mom's issues. I grew up with her trying to commit suicide. I would be the one to take care of her. And I would be the one calling the police to come. It was a lot.

The trauma of the pandemic reminded her, and many others, of the helplessness of childhood. Monique was not alone. Others noted the resurfacing of pandemic‐prompted reflections on parental alcohol misuse. Margaret's experiences influenced her alcohol usage as an adult. She contextualized:

My dad was a big drinker when I was younger. My mom… was a big binge drinker. So that kind of forced me to grow up a lot faster because I was the oldest. [W]hen she started to slow down, her friends would still come around. And they would actually let me drink with them [at the age of 12].

Margaret continued, “Before the pandemic, I would go out with friends. But after the pandemic, I don't do that, I stay home, by myself.” Overall, internalized pandemic stressors contributed to physical and emotional lateral violence and reminded participants of previously experienced trauma and distress.

### 3.3 Physical Symptoms: “We All Had It”

Although many participants highlighted numerous benefits of sharing a home with extended family (e.g., financial and caregiving support), some noted how multifamily homes increased their risk of exposure to the virus. As Molly explained, when one family member contracted the virus, it was difficult to prevent exposure for others:

We all had it in my household. And at that time, it was packed. Like, multiple families. It was my family, my sister and a couple of her kids, and my mom, and then my kids' dad was living with us, too. So… when it first started, we were all living together. We all got it. My mom had it first. And then we just started all getting it.

Work positions and employment often shifted during the pandemic, sometimes increasing COVID‐19 exposure. Kathleen said, “My son is the one that caught it from a coworker.” Kathleen contracted COVID‐19 consequently. Alison thought she contracted COVID‐19 when pursuing a job opportunity at a nursing home. She shared, “I had went to the nursing home to apply for a job. Maybe a day or two after I just started getting real, real, real sick.” Sophia's sister contracted COVID‐19 from her workplace, describing their lack of instituted safety measures, “They're not really doing anything here, you know taking precautions and stuff. They're not doing none [*sic*] of that.”

Preexisting health conditions also increase physical risk for COVID‐19 exposure and illness severity. Michelle shared her uncle passed from COVID‐19 when he was exposed in a health care facility: “[Be]cause of his diabetes… he ended up having to have surgery, but he caught COVID‐19 at the hospital and then ended up passing away from it.” Described physical risk factors, including extended kinships and multifamily homes, frontline work and lack of workplace precautions, and disease comorbidities tended to increase COVID‐19 exposure and severity.

### 3.4 Impaired Unity: “It Actually Tore Our Community More Apart”

For some, pandemic stressors hindered community mindedness and reciprocal sharing of support and resources. Genevieve thought the pandemic “tore our community more apart.” She continued, sharing how what she witnessed during the pandemic differed from stories of past crises:

I love hearing stories about the old time. When I would hear Elders talk about how people in the community will go and help somebody in need… the whole community would come together and cook for them or clean the yard or clean their house for them, while they're worried about that… person. I noticed that I've done it not because I wanted something back. I've done it out of love, I hope they make it through.

Marion mentioned feeling a lack of trust within the community, which impacted community members' willingness to seek help in times of need. She said:

I know [tribal leadership] has offered a lotta [*sic*] services for COVID‐19 but I don't see a lotta [*sic*] people taking that, you know? I think it's just something about going to a hospital… confidentiality. I feel like it's not upheld.

Importantly, Margaret explained how “passed down” historical oppression heightens distrust within families and communities and shared, “[Distrust comes from] being ripped apart from your family and not allowed to go back, and these people are harming you that's supposed to be taking care of you.” Genevieve also felt the pandemic did not bring her community together as it should have, explaining, “I think we've gotten to be so selfish, that we really don't care about other people's needs.” This selfishness was related to alcohol and drug use and put community members at risk, especially Elders. Genevieve shared:

If we cared about somebody else's needs then we wouldn't be doing all these things that we're doing, we want to protect them. Especially our Elders, we lost a lot of our Elders. And they're the backbone to this tribe. And yet, we really didn't protect them.

Marion further characterized this by explaining how some younger tribal members violated curfew while drinking alcohol, exposing Elders when returning home. She said, “There were people out there, jumping houses. They get infected and then they go back home to the grandparents' house, and they get infected and a lot of them lost their life.” This “divided” the community, as family members would blame one another for COVID‐19 exposures.

In general, Janie elaborated, she felt some members of her community did not take COVID‐19 precautions seriously: “They don't wanna [*sic*] wear the mask, they don't wanna [*sic*] vaccinate, it's no big deal to them. I'm like, ‘You haven't lost somebody to the virus, obviously.’” Although the unprecedented circumstances of COVID‐19 prompted individualistic responses from some that put others at risk, Janie concluded, “The only way we're gonna beat this virus is if we all work together.”

### 3.5 Disintegrated Support and Kinship Networks: “Losing Family”

Participants saw a devastating loss of life in a short amount of time, which impacted not only the community at large but also family units and social supports. Sophia thought “losing family” was one of the hardest parts of the pandemic. She made a difficult decision to separate from a family member with a suspected COVID‐19 infection and elaborated on how the virus spread limited loved one's abilities to be together physically:

Family members just getting sick was the hardest part because…you wanted to be there and to care for them and you just couldn't. Because you also had to think about yourself… about your family and your household. That was the hardest part for me is that I couldn't be there to help them.

Similarly, Andrea could not be close to her mother physically when she contracted COVID‐19, saying, “It just broke my heart.”

Social distancing limited gatherings of extended kinships, as exemplified by Luz, who felt “not having… traditional daily activity of anything happening… get‐togethers” was difficult during the pandemic. She further said:

You have families, holidays, birthdays, whatever. You have a big turnout. Now, you didn't during the pandemic… [The Tribal] Chief had sent a memo out… saying no big gathering unless it's only two or six [people] and you have to keep this distance apart from each other.

Sophia contextualized how strong matriarchs often unify families. The loss of such figures to COVID‐19 or otherwise diminishes family plans and gatherings. Sophia shared:

I think when my grandma passed away… all her kids pretty much went to themselves with their family… all the get togethers and all of that stops when… the real mom passes away… So everybody kind of did their own thing.

For Andrea, togetherness or cohesion within the home was impacted negatively by amplified family conflict. Andrea had “angry outbursts out of nowhere” affecting her children and her children would “fight amongst each other” as they got “tired of each other.” Driving anger, she thought “nobody was open‐minded. Everybody just wanted to blame somebody.” She noted the need for connection:

I think everybody was… wanting to be away from each other, stay in the room. So, once they get out, they start fighting… I really don't think we feel connected. I would like to improve it though, somehow.

As these women shared, death, precautionary social distancing, and conflict among family members disintegrated social supports and cohesion previously commonplace.

## 4 Discussion

COVID‐19 profoundly impacted Indigenous health, influenced by sociopolitical, cultural, and historical factors (Hunter et al. 2022). Study participants frequently discussed high death rates in their communities, consistent with existing literature that COVID‐19 worsened preexisting structural and systemic inequities for Indigenous peoples on reservations (Leggat‐Barr et al. 2021). As set forth by the FHORT, participants described patterns of pandemic‐induced harm that followed historic, settler‐colonial devaluing of Indigenous peoples and communities. Inequitable and unequal structural factors contributed to COVID‐19 vulnerability for Indigenous peoples and increased stress at the individual level, cited as overburdening through financial instability, crowded households, employment‐related risk, preexisting disease comorbidities, and strained collectivist systems. Like in participants' stories, Indigenous peoples and other minoritized racial/ethnic groups were more likely to experience frontline work, job instability, and fewer worker protections (Gemelas et al. 2022; Goldman et al. 2021; Leggat‐Barr et al. 2021). Household crowding and multifamily homes were also risk factors for COVID‐19 transmission among Indigenous peoples, correlated with high poverty rates (Fortuna et al. 2020; Leggat‐Barr et al. 2021), yet intergenerational and multifamily homes serve important supportive roles and sociocultural functions that could be protective in crises (Leggat‐Barr et al. 2021). Thomas et al. (2023) posited pathways of how Indigenous peoples embody historical trauma and oppression, causing disproportional rates of comorbid chronic conditions.

Findings corroborate increased microaggressions and discrimination at the interpersonal level, alongside structural inequities and systemic, normalized societal racism. One participant, Andrea, quoted that others in the community were “more vocal” about racism during COVID‐19, an experience corroborated by other participants. Minoritized racial/ethnic groups were more likely to experience pandemic‐related discrimination than their White counterparts, exacerbating preexisting resentment against marginalized communities (Strassle et al. 2022). COVID‐19 occurred concurrently with fear and anxiety, massive unemployment, and national civil unrest over longstanding racial inequities for communities of color. Though dynamics of this social upheaval continue to be explored, heightened experiences of micro‐ and macro‐level racism exposed a “manifestly unequal country” in which longstanding socioeconomic and racial/ethnic divides shape health divides (Galea and Abdalla 2020, 227). Participants described racism and discrimination throughout their lifetimes. These persistent assaults take a considerable toll on Indigenous individuals, families, and communities and maintain racial inequity on a national level (Evans‐Campbell 2008; Senter and Ling 2017).

High COVID‐19 death rates and precautionary physical distancing were not aligned with typical family and community relations. Participants found the inability to physically support or gather with loved ones to be one of the most difficult aspects of the pandemic, particularly if loved ones were sick. Tenets of close‐knit families are often prominent to Indigenous peoples, including “sharing traditions, humor and laughter, and sharing meals and celebrations, along with respect for Elders and all family members” (Burnette et al. 2020, 704). Indigenous family structures frequently include intergenerational households and extension to other households of biological or nonbiological kin (Weaver and White 1997); however, because of COVID‐19 safety precautions, participants could not participate in important extended kinship gatherings, usually facilitated by family matriarchs.

### 4.1 Impaired Collectivism and Colonially Generated Cultural Disruption

COVID‐19 and historical oppression undermine Indigenous values and collectivism. Using the FHORT, historical oppression diminishes Indigenous value systems based on egalitarian, interdependent relationships and aligning oneself with the health of the whole (i.e., all Creation; Blume 2022; Solomon et al. 2022). Internalized oppression refers to the inadvertent adoption of the oppressor's dehumanizing beliefs and behaviors because of chronic and historical oppression. This oppression is then directed laterally and self‐perpetuated within communities and across generations (Burnette and Figley 2017). Alfred (2009) described *colonially generated cultural disruption* among Indigenous peoples of Canada as the extreme limitation of healthy, autonomous communities due to settler–colonial assimilation and the internalization of false, damaging information. From this framework, acts of suboppression and lateral violence, when Indigenous peoples harm or seemingly turn on each other, may be outward demonstrations of deliberate colonial oppression (Alfred 2009; Bailey 2020; McKinley et al. 2020; Poupart 2003). Lack of cohesion and devaluation of vulnerable community members during COVID‐19 should be understood as historical oppression in the form of settler‐colonial cultural disruptions.

Several participants felt the pandemic disrupted community mindedness and promoted individualistic responses that put others' health at risk. The FHORT hypothesizes that internalized oppression mediated the perpetuation of western power constructs, as some forewent collectivism in favor of the colonial assumption of self vs. other (Blume 2022). Moreover, Genevieve commented on the “selfish” acts of community members that particularly hurt Elders, which were antithetical to the honored role of Indigenous Elders that she described as the “backbone to this tribe”; These acts more closely resembled colonial‐induced acculturation that weakened traditional roles and relations. Elders are highly revered traditional knowledge holders who root Indigenous community resilience and healthy future generations (Kahn et al. 2016). Loss of these figures through increased mortality disrupts cultural continuity and the passage of traditional knowledge and wisdom. Women expressed how pandemic‐induced stressors heightened physical and emotional intimate partner violence, with internalized oppression and patriarchal, sexist colonial principles contributing to violence against Indigenous women (Burnette and Figley 2017; McKinley and Liddell 2022). Grim circumstances of COVID‐19 reminded participants of childhood trauma. Participants also shared stories of resilience and survivance, actively resisting settler colonial oppression through cultural identity, relationality, and traditions. Despite COVID‐19 disruptions, Indigenous values and collectivist kinship structures promoted well‐being by fostering community care, safe cultural practices, and positive communication strategies.

### 4.2 Implications

Disproportionate COVID‐19 virus contraction and hospitalization directly threatened Indigenous lives. Just as importantly, sociocultural chronic stressors that predated and worsened during the pandemic increased allostatic load, or stress‐related wear‐and‐tear on various body systems, which has deteriorated physical and psychological health for Indigenous peoples (Thayer et al. 2017). The FHORT allows for nuanced, justice‐oriented understandings of historical and contemporary forms of oppression that strained the well‐being of Indigenous peoples during COVID‐19 while uplifting processes of resilience. Ecosocial/ecosystemic frameworks place health inequities for minoritized persons because of chronic, intergenerational exposure to harmful conditions across individual, interpersonal, communal, and societal levels, driven by policy and political actions (Krieger 2014). This lessens blame on the individual and shifts responsibility to health systems that often fail Indigenous peoples. Public health discourse must recognize settler colonialism as both a historical and present force that disenfranchises Indigenous peoples and burdens them with “mortality and morbidity, inequities entrenched in education and healthcare systems, historic socioeconomic deprivation, and ongoing experiences of violent victimization” (Barcham 2022, 457).

Study participants were highly perceptive of the detriment that compromised collectivist responses had during COVID‐19, resulting in individualism and blaming family and community members. Policy and interventions must support the rights of sovereign tribal nations that historically have been undermined, including self‐governance and reconnection to ancestral lands and cultural identity. Many Indigenous knowledge systems and practices provide an integral basis for well‐being in public health disasters, such as land reliance, Indigenist foodways, and relationships with the environment and one another (Elliott‐Groves et al. 2020; McKinley and Walters 2023). Liberating such strengths allows Indigenous peoples to care for their own communities and adapt during catastrophic change.

### 4.3 Limitations

There are limitations to the generalizability of this study's findings regarding the broader Indigenous experience of COVID‐19 in the U.S. Indigenous peoples and communities are diverse, and this study reflects the views of only a subset of female heads of households from a specific tribal community. This study also did not specifically examine differences in themes based on participant age or relationship status. While these factors may influence family and community experiences, our analysis focused on broader themes emerging from participants' narratives. Future research could explore these variables to provide deeper insight into potential variations in responses based on demographic characteristics. Additionally, the retrospective nature of the interviews may have led participants to underreport their experiences since the onset of the pandemic and their ongoing challenges. This research focuses on risk factors affecting these participants' COVID‐19 experiences, but it underrepresents the numerous stories of strength and resilience amid systemic oppression. Further research on protective factors following extreme hardship is needed.

## 5 Conclusion

The findings demonstrate that ongoing historical oppression exacerbates the collective health of Indigenous peoples and undermines well‐being at all ecological levels. Additionally, this study revealed that COVID‐19‐related risk factors primarily stem from systemic inequities, the incongruence between Indigenous family systems and physical distancing, and weakened collectivism. These results highlight the need for crisis interventions that align with Indigenous values and ways of knowing, promoting traditional protective factors consistent with the FHORT (Burnette and Figley 2017). Further research is necessary to enhance our understanding of the protective factors that effectively mitigate the diverse impacts of COVID‐19 and how professionals can best integrate and promote this knowledge in future interventions.

## Funding

This work was supported by the National Institute on Alcohol Abuse and Alcoholism of the National Institute of Health under Award Number R01AA028201 and the National Institutes of Health NOT‐MH‐20‐053, Notice of Special Interest (NOSI): Digital Healthcare Interventions to Address the Secondary Health Effects Related to Social, Behavioral, and Economic Impact of COVID‐19 under Grant 3R01AA028201‐01S1.

## Conflicts of Interest

The authors declare no conflicts of interest.

## Data Availability

Research data are not shared.
